# Aberrant epigenetic regulation in clear cell sarcoma of the kidney featuring distinct DNA hypermethylation and EZH2 overexpression

**DOI:** 10.18632/oncotarget.7152

**Published:** 2016-02-03

**Authors:** Jenny Karlsson, Anders Valind, Caroline Jansson, Maureen J. O'Sullivan, Linda Holmquist Mengelbier, David Gisselsson

**Affiliations:** ^1^ Department of Clinical Genetics, Lund University, University and Regional Laboratories, Lund, Sweden; ^2^ National Children's Research Centre, Our Lady's Children's Hospital, Crumlin, Dublin, Ireland; ^3^ Department of Pathology, Skåne Regional and University Laboratories, Lund, Sweden

**Keywords:** CCSK, Wilms tumor, pediatric tumors, hypermethylation, EZH2

## Abstract

The global methylation profile and the mutational status of 633 specific epigenetic regulators were analyzed in the pediatric tumor clear cell sarcoma of the kidney (CCSK). Methylation array analyses of 30 CCSKs revealed CCSK tumor DNA to be globally hypermethylated compared to Wilms tumor, normal fetal kidney, and adult kidney. The aberrant methylation pattern of CCSKs was associated with activation of genes involved in embryonic processes and with silencing of genes linked to normal kidney function. No epigenetic regulator was recurrently mutated in our cohort, but a mutation in the key epigenetic regulator *EZH2* was discovered in one case. *EZH2* mRNA was significantly higher in CCSK compared to Wilms tumor and normal kidney, and the EZH2 protein was strongly expressed in more than 90 % of CCSK tumor cells in 9/9 tumors analyzed. This was in striking contrast to the lack of EZH2 protein expression in Wilms tumor stromal elements, indicating that EZH2 could be explored further as a diagnostic marker and a potential drug target for CCSK.

## INTRODUCTION

Clear cell sarcoma of the kidney (CCSK) is the second commonest pediatric renal cancer, surpassed only by Wilms tumor (WT) [1]. WT and CCSK most often affect children younger than 5 years. CCSKs are more prone to form metastases than WT, particularly to brain and bone and are hence treated according to high-risk protocols, including combinations of traditional chemotherapeutic substances. There is therefore an urgent need for more specific treatment modalities, associated with fewer side effects [1]. CCSKs typically display a normal karyotype even when subjected to high-resolution genome array analysis [2, 3]. A subgroup of CCSKs has been shown to contain specific gene fusions including *YWHAE-NUTM2B/E* and *IRX2-TERT* [4, 5], but these fusions are rare and their clinical implications remain unknown. Recently, targeted sequencing showed that an internal tandem duplication (ITD) in exon 16 of the *BCOR* gene, is present in the majority of CCSKs, while typically not in those carrying the *YWHAE-NUTM2B/E* fusion [6, 7]. On the other hand, a comprehensive molecular characterization of a small set of CCSKs failed to detect any somatic genetic variants [8], reinforcing the consideration that CCSKs are relatively quiescent also at the DNA sequence level.

We previously reported that CCSK likely emerges from embryonic mesenchymal progenitor cells at an earlier stage of nephrogenic differentiation than the cells giving rise to WT [3]. Interestingly, BCOR has been shown to have a prominent role in the maintenance of differentiation in mesenchymal stem cells through epigenetic mechanisms [9]. BCOR is a component of the non-canonical polycomb repressive complex 1 (PRC1) [10, 11], which marks histones for the recruitment of polycomb repressive complex 2 (PRC2)-complexes [12]. BCOR has also been shown to affect local histone acetylation and methylation states as well as DNA methylation [13, 14]. The role of BCOR as an epigenetic regulator together with the striking paucity of large genomic imbalances, and the few detected somatic mutations suggests that the primitive mesenchymal expression profile of CCSK results mainly from epigenetic dysregulation.

Here we explore the epigenetic profile of CCSK by analyzing the DNA methylation pattern of 30 cases and the mutational status of 633 epigenetic regulators in 22 cases of this tumor. We find that CCSK exhibits a characteristic DNA methylation profile, but fail to find mutations of epigenetic regulator genes. Interestingly, we found a high expression of the key epigenetic regulator protein EZH2 in all CCSKs tested, which could have future clinical implications.

## RESULTS

### CCSK displays a distinct global DNA methylation profile

**Figure 1:**
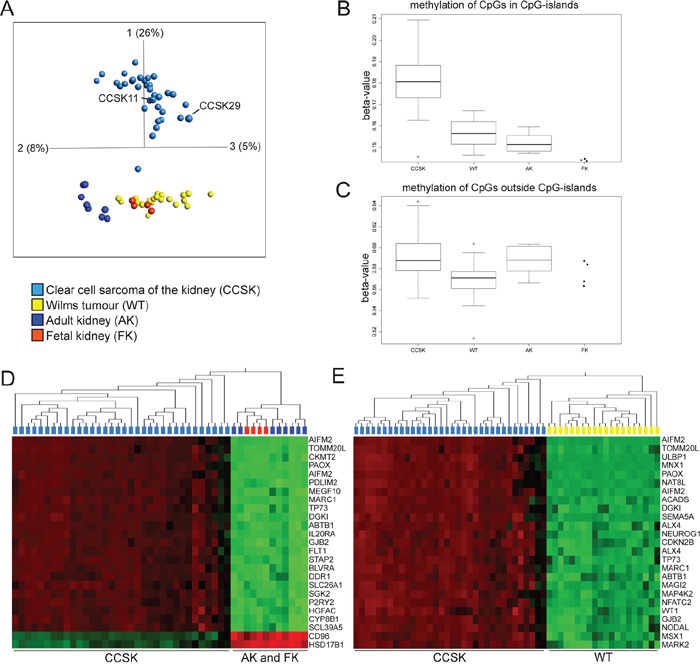
CCSKs display global hypermethylation of CpGs located in CpG-islands **A.** Principal component analysis of DNA methylation (0.05 variance ratio, 15051 variables) of 30 CCSKs, 17 WTs, 6 adult kidneys (AK), and 2 fetal kidneys (FK). Four of the CCSKs and two WTs, AKs, and FKs were analyzed in duplicates on different arrays. CCSK11 and CCSK29 harbor the YWHAE-NUTM2B/E fusion transcript. **B** and **C.** Boxplots based on the median beta-value for CpGs **B.** situated in CpG islands and **C.** outside CpG-islands. **D** and **E.** The 25 genes associated with the most variably methylated CpGs after ANOVA based hierarchical clustering analyses. **D.** CCSK in relation to adult and fetal kidney (p-value = 5.9×10^−27^) and **E.** CCSK in relation to WT (p-value = 3.0×10^−28^). Variables with high methylation are depicted in red and low in green. The variables are sorted according to fold change with the most variably methylated CpGs at the top of the heat map.

Array-based DNA-methylation analysis revealed that the global methylation profile of CCSKs was clearly distinct from WTs as well as non-neoplastic fetal and adult kidneys (Figure 1A). The two tumors (CCSK11 and CCSK29) previously shown to harbor the *YWHAE-NUTM2B/E* fusion [7] clustered with the fusion negative and *BCOR* ITD positive tumors, in the principal component analysis (PCA) plot. WTs clustered together with normal kidneys, and their methylation status resembled fetal kidney to a greater extent than adult kidney. Overall DNA methylation, as reflected by the median beta-value for all probes, was higher in CCSK than in the other sample sets. Separate analyses of the methylation status of CpGs situated in or outside of CpG islands (Figure 1B and 1C), revealed that it was CpGs located within CpG islands that were significantly hypermethylated in CCSK compared to WT (p-value = 2.3×10^−9^), adult kidney (p-value = 5.7 x10^−6^), and fetal kidney (p-value 0.0001; Figure 1B). There was no significant difference between mean methylation levels of CpGs located outside CpG islands in CCSK and non-neoplastic kidneys (Figure 1C), indicating that the aberrant hypermethylation of CCSKs is confined to CpG islands.

We then established which methylated gene regions primarily discriminated between CCSK on the one hand, and normal kidney or WT on the other (Figure 1D and 1E). As expected, the majority of these differentially methylated CpGs displayed a higher beta-value in CCSK. Of the 25 most differentially methylated regions, eight genes (*TP73, GJB2, ABTB1, DGK1, PAOX, MARC1, AIFM2* and *TOMM20L*) were hypermethylated in CCSK in comparison with both normal kidney and WT (Figure 1D and 1E). Gene set enrichment analysis (GSEA) revealed that 241 pathways were significantly over-represented among the hypermethylated genes in CCSK compared to WT, adult kidney, and fetal kidney, while no gene sets were significantly over-represented amongst hypomethylated genes in CCSK compared to WT. Hypermethylated genes in CCSK were most significantly associated with biological pathways involved in cell adhesion, anatomical structure morphogenesis, organ development, and cell migration (Table 1 and Supplementary Table 1). Corresponding pathways were enriched if the CCSK methylation profile was compared to normal kidney or WT separately (data not shown), in line with the similarities in methylation profile of these tissue types (Figure 1A). Hypermethylated genes in WT, fetal or adult kidney did not significantly correlate with any gene set irrespective of comparison group (data not shown).

**Table 1:** Enriched signaling pathways among hypermethylated genes in CCSK compared to WT and non-malignant kidney tissue, (nominal p-value < 0.001, FDR q-value < 0.2)

PATHWAY	NES^a^	FDR q-value^b^
CELL-CELL ADHESION	1.75	0.10
ANATOMICAL STRUCTURE MORPHOGENESIS	1.73	0.08
GLYCEROPHOSPHOLIPID BIOSYNTHETIC PROCESS	1.73	0.08
ORGAN DEVELOPMENT	1.66	0.11
CELL MIGRATION	1.63	0.10

a NES-Normalized enrichment score

b FDR q-value false discovery rate

### The aberrant methylation pattern in CCSK is linked to activation of embryonic genes and silencing of genes important for kidney function and epithelialization

**Figure 2:**
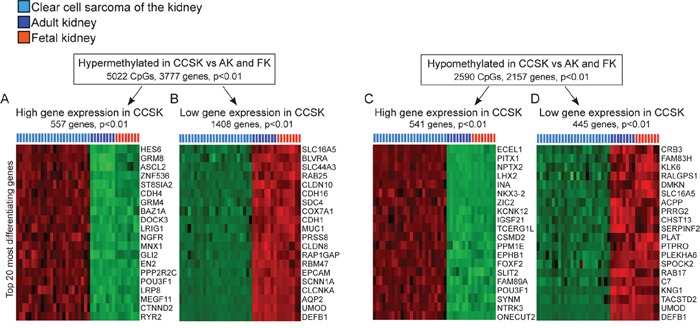
Methylation directed gene expression in CCSK Genes with hyper- **A** and **B.** and hypomethylated CpGs **C** and **D.** in CCSK compared to non-neoplastic fetal and adult kidney were extracted (p< 0.01). The expression pattern of these genes in CCSK and the normal kidney samples was identified, and significantly (p< 0.01) up-regulated **A** and **C.** or down-regulated **B** and **D.** genes in CCSK were extracted. The 20 genes that differentiated CCSK the most from normal kidney are displayed according to fold-change, with the most variably expressed gene at the top of the heatmaps. Variables with high expression are shown in red and low in green.

To monitor the potential functional effects of the unique DNA methylation signature of CCSK, we identified genes with differential mRNA expression that localized to hypo/hypermethylated regions in CCSK compared to adult and fetal kidney (Figure 2). 5022 CpGs were significantly (p< 0.01) hypermethylated in CCSK in 3777 unique genes (Figure 2A and 2B) and 2590 CpGs localised to 2157 unique genes were hypomethylated (Figure 2C and 2D).

The gene expression patterns of the extracted hyper- and hypomethylated genes were investigated with gene expression array. This showed an enrichment for genes involved in embryonic development and organ formation amongst those with high expression in CCSK irrespective of whether the expression was associated with hypermethylation in CCSK as exemplified *HES6, CDH4, ASCL2, GLI2, EN2, LRP8*, and *CTNND2* (Figure 2A) or hypomethylation e.g. *PITX1, NPTX2, LHX2, NKX3, ZIC2, SLIT2, NTRK3, ONECUT2*, and *EPHB1* (Figure 2C). Among genes where high methylation was linked to a silenced gene expression in CCSK, there was an enrichment of genes associated with normal kidney formation (*AQP2* and *UMOD*) and genes typically expressed by epithelial cells (*EPCAM, SCNN1A, CLDN8*, and *CDH16)* (Figure 2B). Genes with low methylation and low expression in CCSK were not associated with any specific signaling pathway (Figure 2D).

### Absence of recurrent mutations in epigenetic regulators in CCSK

To investigate a possible underlying mechanism behind the unique signature of DNA hypermethylation in CCSK, we used our previously published RNA-sequencing data from 22 of these tumors [4] to screen for single nucleotide variants (SNVs) in 633 genes previously identified as epigenetic regulators [15]. No genes were recurrently mutated, but exonic nonsynonymous variants were identified in 14 genes in 50% (11/22) of the analyzed tumors, all of which were verified by deep DNA sequencing (Table 2). As we called variants using RNA-sequencing data and had no paired normal samples, we employed strict criteria for calling variants (see Materials and Methods for details). The mutation in CCSK21 in *EZH2* was the only variant previously reported in the COSMIC database (COSM52996) [16].

**Table 2:** Mutations in epigenetic regulators in CCSK identified by deep targeted sequencing of tumor genomic DNA

SAMPLE	GENE	CHR	POSITION	REF/ALT	VAF^a^	TYPE
CCSK5	RUVBL1	3	127816147	T/C	0.47	nonsynonymous SNV
CCSK9	MEAF6	1	37959388	C/T	0.47	stopgain
CCSK11	H2AFX	11	118966104	T/A	0.48	nonsynonymous SNV
CCSK15	MBD6	12	57922317	C/T	0.54	nonsynonymous SNV
CCSK19	ASH2L	8	37963213	G/T	0.46	nonsynonymous SNV
CCSK21	BAZ1A	14	35234247	A/T	0.55	nonsynonymous SNV
CCSK21	EZH2	7	148525838	G/A	0.53	stopgain
CCSK25	PHF20L1	8	133844572	A/T	0.47	nonsynonymous SNV
CCSK28	PHC2	1	33790591	C/T	0.52	nonsynonymous SNV
CCSK30	KDM5D	Y	21877281	A/G	0.99	nonsynonymous SNV
CCSK31	KDM5C	X	53227048	C/T	0.99	nonsynonymous SNV
CCSK31	BABAM1	19	17379817	G/A	0.48	nonsynonymous SNV
CCSK31	ARID1A	1	27097755	C/G	0.50	nonsynonymous SNV
CCSK37	SMC1A	X	53409246	C/T	0.92	nonsynonymous SNV

a VAF- variant allele frequency

### EZH2 is highly expressed in CCSK

**Figure 3:**
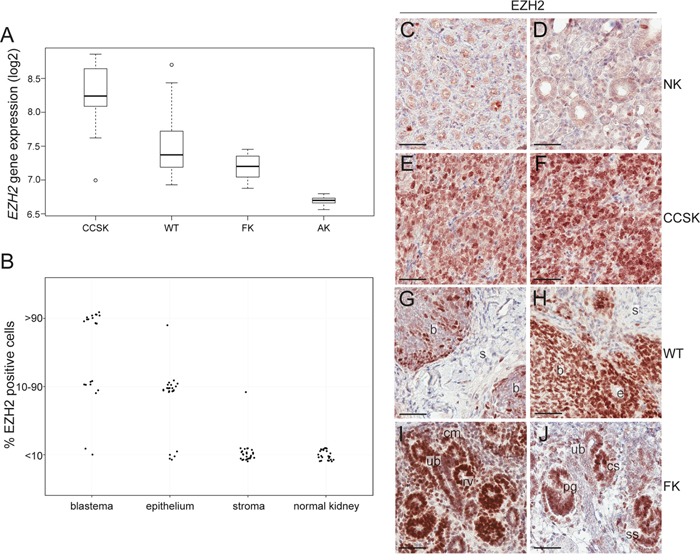
EZH2 is highly expressed in CCSK **A.** Boxplots of the median gene expression level of *EZH2* in clear cell sarcoma of the kidney (CCSK), Wilms tumor (WT), fetal kidney (FK), and adult kidney (AK). **B.** The proportion of EZH2-positve cells in the different histological elements of 30 WTs and adjacent non-neoplastic tissue was evaluated. The different histological elements were categorized based on the amount of EZH2-expressing cells (less than 10%, 10-90% or more than 90% positivity). **C-J.** Immunohistochemistry demonstrating the EZH2 protein expression pattern in **C** and **D.** normal kidney, **E** and **F.** CCSK, **G** and **H.** WT, **I.** FK gestational week 12-13 and **J.** FK gestational week 19-20. C/E and D/F, respectively, are from the same CCSK patients. b= blastema, s= stroma, e= epithelium, cm= cap mesenchyme, ub= ureteric bud, rv = renal vesicle, cs = comma shaped structure, ss= s-shaped structure, pg= primitive glomerulus. The scale bar represents 50 μm.

When investigating the gene expression level of the 633 epigenetic regulators using gene expression array data, we found the key epigenetic regulator *EZH2* to be significantly higher expressed in CCSKs compared to WTs, fetal kidneys, and adult kidneys (Figure 3A). *EZH2* was highly expressed in CCSKs irrespective of whether the tumour harboured the *BCOR*-ITD or the *YWHAE-NUTM2B/E* fusion transcript (not shown). The median *EZH2*-expression levels were similar between WTs and fetal kidneys, while adult kidneys displayed the lowest *EZH2*-expression. The protein expression of EZH2 was strong and diffuse (>90% positive cells) in 9/9 CCSKs analyzed (Figure 3E and 3F). All round or spindle shaped tumor cells strongly expressed EZH2, in contrast to the cells in the fibrous septa and the vasculature of the tumors, which were EZH2-negative. Adjacent normal kidney tissue was EZH2-negative (Figure 3C and 3D), with only occasional EZH2-positive cells in renal tubules.

In WT, the protein expression of EZH2 differed between the different histological components (Figure 3B, 3G, and 3H). The stromal component stained negative/weak (<10% positive cells) in 26 of the 27 WTs with stromal areas represented. Widespread stromal expression was detected only in an unusual WT with anaplastic stromal histology (not shown). Twenty-four of the WTs analyzed harbored epithelial components, and the majority of the tumors were EZH2-positive in 10-90% of these epithelial cells. Two different staining patterns were detected for the 23 WTs analyzed which contained blastemal components; EZH2 was positive in 10-90% of the blastemal cells in 7 cases (Figure 3G), while almost all (>90% positive cells) blastemal cells were strongly and diffusely positive in 14 of the tumors (Figure 3H). As an incidental observation, we noted that none of the WT patients with <90% blastemal EZH2 positivity died from their disease, while 7 out of the 14 patients with widespread blastemal EZH2 - positivity were dead of disease or complications (Supplementary Table 2). This suggests a possible correlation between diffuse blastemal EZH2 protein expression and less favorable prognosis (p = 0.0189, Fisher's exact test).

Analogous to the findings in WTs, fetal kidney showed an expression of the EZH2 protein, indicative of a role in early nephrogenic elements. The expression was widespread at gestational week 12-13 (Figure 3I), but became specific for epithelial structures in the more differentiated kidney from gestational week 19-20 (Figure 3J).

## DISCUSSION

In summary, we found that CCSKs display a distinct DNA methylation profile compared to WT and normal kidney tissue. The global methylation pattern of the WTs on the other hand was similar to non-neoplastic kidney, especially fetal kidney. Specifically, CpGs localized to CpG islands were aberrantly methylated in CCSK. Previous studies have indicated that CCSKs are characterized by disturbed embryonic signaling pathways [3, 17]. In the present study, we found a clear link between the aberrant DNA methylation pattern in CCSK and the expression of genes important in embryonic pathways and cellular organization. This indicates that the characteristic gene expression pattern of CCSK is, at least in part, determined by the methylation of specific genes. Together with the recent finding of a recurrent BCOR-ITD in CCSK [6], our data highlights the significance of epigenetic dysregulation in CCSK.

It is well established that an aberrant hypermethylation of tumor DNA can be caused by mutations in epigenetic modifiers such as *IDH1, IDH2*, *TET1, TET2*, and *SDH*-subunits [18, 19]. Hence we analyzed RNA sequencing data from a previous study [2] to search for mutations in 633 genes identified as epigenetic regulators with some of them commonly mutated in pediatric tumors [15]. We did identify deleterious mutations in 50% of tumors analyzed, but no gene was recurrently mutated in our panel of tumors. Due to the lack of non-malignant material from the patients, we cannot rule out the possibility that some of the identified aberrations are rare constitutional variants. On the other hand, our findings make it unlikely that other recurrent driver mutations besides the *BCOR*-ITD are present in CCSK.

We discovered a previously reported deleterious mutation in *EZH2* (COSM52996) in one of the tumors and we also found high expression of EZH2 in CCSK at both mRNA and protein levels. EZH2 is overexpressed in several solid tumor types, and high expression is correlated with aggressive disease in specific cancers [20]. High expression of EZH2 has been demonstrated in small cell lung cancer, another tumor characterized by hypermethylation of promoter regions [21]. EZH2 is an epigenetic regulator and is the catalytic subunit of the PRC2 which methylates histone 3 at lysine 27 (H3K27). The trimethylated form of this complex represses gene expression during differentiation and development [22]. EZH2 also has a PRC2-independent function and can activate expression of cell cycle associated genes such as *CCND1* [23], which is in line with the high expression of cyclin D1 previously observed in CCSK [24]. The expression of EZH2 has previously been shown to decrease upon kidney maturation in the mouse fetal kidney [25]. We noted a parallel pattern in the human fetal kidney; EZH2 was broadly expressed at the earliest investigated time point (gestational week 12-13), but was confined to the ureteric bud and to primitive glomerular structures in the more mature kidney (gestational week 19-20).

Together with the recently identified frequent *BCOR* mutations in CCSK [6], our data underscore that epigenetic dysregulation is central to CCSK pathogenesis. An interesting avenue to explore would be a potential connection between the *BCOR*-ITD and EZH2 overexpression, and their potential roles in the global hypermethylation of CCSK reported in the current paper. The lack of EZH2 expression in WT stromal elements renders EZH2 a candidate diagnostic marker to distinguish stromal type WT from CCSK. It is also interesting that strong and diffuse expression of EZH2 in the blastemal compartment correlates with poor prognosis in WT, but these data requires validation in a larger patient cohort. Our findings of high EZH2 protein expression in all investigated CCSKs could be of potential therapeutic interest, considering the recent emergence of several EZH2 inhibitors, with promising anti-tumorigenic effects [20, 21, 26].

## MATERIALS AND METHODS

### Tumor and kidney samples

The study was reviewed and approved by the regional ethics committee (L119-03) and by the review boards of the participating institutes. Frozen tissue from CCSKs was obtained from Children's Oncology Group (COG) of North America, and from Skåne University Hospital, Lund, Sweden. WTs were from Skåne University Hospital and from the Academic Medical Center, Amsterdam, the Netherlands. See Supplementary Tables 1 and 2 for clinical data. CCSK tissue sections were acquired from Our Lady's Children's Hospital, Crumlin Dublin 12, Ireland and from Skåne Regional and University Laboratories, Lund, Sweden. Frozen non-neoplastic kidney tissue was obtained from the biobank at the Department of Pathology at Lund University (Ethics Approval L2012-405). DNA from human fetal kidneys were acquired from BioChain, Hayward, CA.

### Methylation array

The global methylation profile of 30 CCSKs, 17 WTs, six samples from adult kidneys, and two human fetal kidneys were analyzed by methylation array. Four of the CCSKs and two of the WTs, as well as adult kidneys and fetal kidneys were analyzed in technical duplicates (Supplementary Table 3). DNA was extracted from fresh frozen tissue with the DNeasy Blood & Tissue Kit (Qiagen, Valencia, CA), according to the manufacturer's recommendations, and subjected to bisulfite conversion with the EZ DNA methylation kit. DNA was hybridized to the Infinium HumanMethylation27 Bead array (Illumina) at the Swegene Centre for Integrative Biology at Lund University (SCIBLU) according to standard procedures. This array covers 27,578 individual CpG sites in 14,495 genes. The majority of the reporters (20,006) correspond to CpGs located in CpG-islands. The remaining 7,572 reporters correspond to CpG loci that do not meet the CpG island criteria according to Takai and Jones [27]. The methylation value for each CpG, the beta-value, was calculated as the ratio between the signal intensity detected by a methylation-sensitive probe and the sum of the signal intensities detected by the methylation and non-methylation sensitive probes. Hence the beta-values ranged from 0 (unmethylated) to 1 (fully methylated). The samples were analyzed in three different batches consisting of two bead chips in each batch. To adjust for batch effects we used the ComBat implementation in the R/Bioconductor package sva [28]. The chip number was used as batch covariate, and the tissue type was used as the sole biological covariate. The samples clustered according to tissue type and not chip or array after applying this algorithm (Supplementary Figure 1), and duplicates analyzed on different arrays did co-localize after PCA. Probes monitoring non-autosomal loci were removed to adjust for gender differences prior to data analyses. The methylation data are available at the NCBI's Gene Expression Omnibus database, http://www.ncbi.nlm.nih.gov/geo/, accession number GSE73187.

### Descriptive analyses on methylation and gene expression data

Dendrograms were generated using hierarchical clustering (R function hclust), with Euclidean distance as the distance metric. Boxplots were done for each tumor type using the per sample mean. Student's t-test was used to assess whether there was a significant difference in mean methylation between groups. PCA and hierarchical clustering (HCL) with heat maps displaying the methylation and gene expression levels for the individual samples were done with Qlucore Omics Explorer Software v3.0 (Qlucore AB, Lund, Sweden). Each variable was normalized to mean 0 and variance 1. HCL was done using the Euclidean metric and the Average linkage criteria. Gene expression array data were log2-transformed prior to analyses.

The correlation of hyper- or hypomethylated genes in CCSK to specific signaling pathways was investigated by GSEA with the c5.bp.v5.0.symbols.gmt file http://www.broad.mit.edu/gsea/msigdb according to the manufacturer's instructions [29].

### Identification of genes with a plausible methylation-directed expression

Genes with high and low methylation status in all CCSKs in comparison with normal kidney (fetal and adult), were identified with t-test, two group comparison, p < 0.01 (Qlucore). The expression levels of the aberrantly methylated genes were investigated using previously published gene expression array data, HumanHT-12 v4 Expression BeadChips, Illumina [4], public in the Gene Expression Omnibus database, http://www.ncbi.nlm.nih.gov/geo/, GSE49972. Differentially expressed genes in CCSK compared to normal kidney were identified as above.

The gene expression data were merged using the Combat algorithm [28] to another data set (the Gene Expression Omnibus database, http://www.ncbi.nlm.nih.gov/geo/, GSE73209, including 32 WTs in addition to eight samples (two CCSK, two adult kidneys and four fetal kidneys) also analyzed on the CCSK gene expression array described above. The RNA from the WTs was extracted as previously described [3] and hybridized to HumanHT-12 v4 Expression BeadChips. For sample details see Supplementary Table 3.

### Identification of single nucleotide variants from RNA-sequencing data

With the RNA-sequencing data previously described [4], we called single nucleotide variants with the RVBoost pipeline [30]. In short, RVBoost calls variants using the UnifiedGenotyper tool from the GATK suite and annotates variants with a QScore value. Six different sequence features contribute with information to the QScore, facilitating robust annotation of variants introduced by the transcriptional apparatus and cDNA-synthesis. Variants that were annotated by RVBoost as putative RNA-editing events and variants covered by less than 10 reads were removed together with variants with a QScore below 0.1. Variants were also removed if present at any frequency in dbSNP version 130, the 1000 Genomes October 2014 release or release 0.3 of the ExAC dataset from the Exome Aggregation Consortium (ExAC), Cambridge, MA (http://exac.broadinstitute.org) accessed June, 2015. Only variants present in the coding regions of the 633 epigenetic regulators [22] were retained for visual inspection using the Integrative genomics viewer, http://www.broadinstitute.org/igv/ [31]. Non-synonymous variants detected by more than 40% of the reads were validated by amplifying an approximately 300 bp long DNA segment covering the nucleotide of interest with primers designed with Primer 3, http://bioinfo.ut.ee/primer3/. Primer sequences are listed in Supplementary Table 4. The size and relative concentration of the PCR-products were established with agarose gel electrophoresis and equal amount of the different amplicons were pooled and diluted. DNA was prepared for sequencing with the Nextera XT DNA sample preparation kit and Index kit (Illumina) according to the manufacturer's recommendation and sequenced on a HiScanSQ (Illumina).

### Immunohistochemistry and tissue microarray

Formalin-fixed, paraffin embedded (FFPE) sections (4 μm) were deparaffinized in xylene and rehydrated to water through graded alcohols. Endogenous peroxidase was blocked with 1% H_2_O_2_ (Sigma-Aldrich) diluted in PBS pH 7.4 (Applichem) for 20 min. Heat induced epitope retrieval (HIER) was performed with Target Retrieval Solution pH 9.0 (DAKO) and 0.2 % Triton-X100 (Sigma) in a pressure cooker at 120°C for 20 min. Sections were incubated with primary antibody EZH2 1:400 (ab191080, Abcam) diluted in PBS containing 5% normal goat serum (Jackson Immuno Research) for 60 min. Staining was visualized using BrightVision Poly-HRP-Anti rabbit RTU (AH Diagnostics) for 30 min, followed by incubation with DAB (Liquid DAB+ Substrate Chromogen System, DAKO) for 5 min, and counterstained with Mayers Htx (Histolab) for 30 sec. Slides were mounted with Faramount Aqueous Mounting Medium (DAKO). A tissue microarray with cores from the different histological compartments in totally 33 primary WTs [32] was used to investigate EZH2-protein expression.

## SUPPLEMENTARY FIGURE AND TABLES








