# Supplementary material for: Aberrant epigenetic regulation in clear cell sarcoma of the kidney featuring distinct DNA hypermethylation and EZH2 overexpression
Source: Oncotarget. 2016 Feb 3;7(10):11127–36. doi: 10.18632/oncotarget.7152 (PMC4905462; doi:10.18632/oncotarget.7152)
Supplement: Supplementary file 1 [file oncotarget-07-11127-s001.pdf]

SUPPLEMENTARY FIGURE AND TABLES

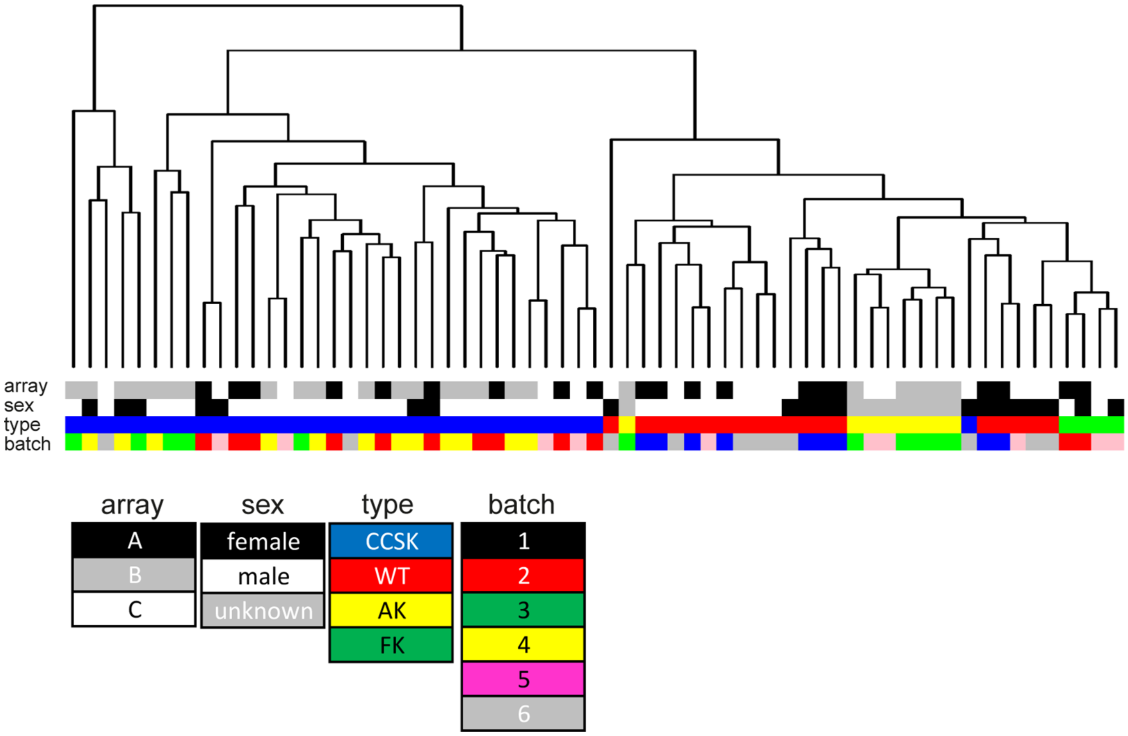

Supplementary Figure S1: Dendrogram of methylation array data after batch effect removal. The dendrogram shows that the samples do not cluster according to chip or batch, but instead they cluster depending on tissue type. Furthermore, the biological replicates cluster tightly, reinforcing that the batch effect adjustment using combat was successful in removing the majority of the batch effects.

Supplementary Table S1: Enriched signalling pathways associated with highly methylated genes in CCSK compared to Wilms tumor, normal adult kidney and normal fetal kidney (FDR q-val < 0.2)

See Supplementary File 1

Supplementary Table S2: Evaluation of EZH2 protein expression in different histological compartments in Wilms tumor. If the histological component was present in the specific tumor, the staining pattern was scored as 0 (< 10% positive cells), 1 (10-90% positive cells) or 2 (> 90% positive cells)

See Supplementary File 2

Supplementary Table S3: Case information

See Supplementary File 3

Supplementary Table S4: Primers used for validation of SNVs in CCSK

Primer name	Primer sequence (5'-3')
H2AFX_f	CGGTGAGGTACTCCAGCACT
H2AFX_r	GTGTGTCCTGGGGGCTTAT
PHC2_f	CCCTAGGAGTCCTTGAGCAT
PHC2_r	CAAGGCGTGGTTATGAGGAC
KDM5D_f	AACAATTCTCCTGCCTCAGC
KDM5D_r	TGCTAACACTGCCTACCTGTTG
SMC1A_f	TCTCTTCGTCAACTGCCCTA
SMC1A_r	GCTTCCTGTCCCTATCTCCA
RUVBL1_f	AACAACATGACCTCCCCTCA
RUVBL1_r	GGGTGTGCTGTTTGTTGATG
MEAF6_f	CAACAAGAGAAAAACAAGAAAACA
MEAF6_r	TCATTTCTGTAAGACATTGGGTCT
MBD6_f	CCCTCAAATGGGGGACAC
MBD6_r	GATGGACTATGTGTGTGGGAAA
PHF20L1_f	TGACTTTCGTTATTTTTATAGTGACC
PHF20L1_r	TTACACATTTAGGAATTGTAAGTCTTG
KDM5C_f	ATCCCTCATCAGCACTCCAA
KDM5C_r	CGAGATCAAGGTGGACAGGT
ARID1A_f	GGGAACTTGCAACCAACCT
ARID1A_r	GAATAAGAGGCCAGGGCAAC
BABAM1_f	CACTCGCTCCAATCCTGAAG
BABAM1_r	CAAAGGGAATGTTTGCAGTG
ASH2L_f	CGCGCGAGAGAAGAGAGTAT
ASH2L_r	ATAACGACAGGAGCCTCAGC
EZH2_f	CCACCCTACCTGGCCATAAT
EZH2_r	ATAAATGATGAAATTTTTGTGGAGT
BAZ1A_f	CAGTCTCCTTCAGGCACAGTC
BAZ1A_r	TGGATCGTAGCGTGATATGG
